# Why Don’t They Want to Wear Sunscreen? An Observational Study of Anti-Sunscreen Messaging on TikTok

**DOI:** 10.1177/10732748261476838

**Published:** 2026-08-12

**Authors:** Carla Herman, Nathalie Harb, Meghri Ghazarian, Eric Belzile, Nina Morena, Ari N. Meguerditchian

**Affiliations:** 1 Faculty of Medicine and Health Sciences, 12367 McGill University, Montreal, Canada; 2 Faculty of Medicine, 12370 Université de Sherbrooke, Sherbrooke, Canada; 3 Department of Journalism, 5618 Concordia University, Montreal, Canada; 4 St Mary’s Research Centre, Montreal, Canada; 5 Department of Art History and Communication Studies, Faculty of Arts, 12367 McGill University, Montreal, Canada; 6 Departments of Surgery & Oncology, McGill University Health Centre, 5620 McGill University, Montreal, Canada

**Keywords:** melanoma, skin cancer, TikTok, social media, sunscreen

## Abstract

**Introduction:**

The incidence of melanoma among younger patients is increasing. This demographic often turns to social media for information and connection. This observational study aims to quantify the prevalence of anti-sunscreen sentiments on TikTok and analyze their content and communication style.

**Methods:**

Videos tagged #nosunscreen on TikTok were collected in July 2024. Reviewers gathered video characteristics such as username, user profile (individual or organisation), date posted, captions, length, number of followers, likes, views, shares, and comments, presence of sponsorship, and filming location. User demographics were noted. Reviewers also noted communication styles and other video characteristics such as the use of audio memes or music. Users’ opinions about not wanting to wear sunscreen were collected and analysed. Spearman’s correlation coefficient, Chi-squared test, and Phi coefficient were calculated between variables.

**Results:**

321 English language TikToks were selected. The majority (38%) were posted in 2023. Average video length was 25 seconds. Mean number of followers was 90,053 (SD 324,640). Mean number of views, likes, and comments was 73,121 (SD 408,245), 5,250 (SD 31,772), and 51 (SD 203), respectively. Only 12 videos included product sponsorship. Female (78%) predominance was observed and 19% showed a person belonging to a visible ethnic minority. 45% of videos used music and 30% used audio memes. 32% were filmed outdoors, mostly the backyard or the beach. In 44% of videos, a sunburn was shown. Almost half of the videos (41%) provided explicit sunscreen sentiments. Common reasons for not wearing sunscreen included personal preferences, trends, perceived natural sun exposure benefits, sunscreen being viewed as unnecessary and/or harmful, alternative forms of sun precaution, and the desire to tan. 42% of videos conveyed sarcasm and/or irony. Anti-sunscreen stances were strongly associated with a lack of regret for not wearing sunscreen (p<0.001).

**Conclusion:**

TikTok videos tagged with #nosunscreen highlight the alarming prevalence of anti-sunscreen sentiments.

## Introduction

The incidence of melanoma among younger populations has been steadily rising in North America,^1-4^ posing a significant public health concern. Early life exposure to ultraviolet radiation and a history of sunburn during youth have been shown to increase the risk of skin cancer.^5-7^ This underscores the importance of implementing effective sun safety strategies, such as limiting sun exposure and using sunscreen.^8,9^ Despite skin cancer being a preventable malignancy and widespread public awareness campaigns, sunscreen use remains alarmingly inconsistent among younger people,^10,11^ leaving them at risk of a largely preventable disease.

Social media represents the dominant source of information for younger demographics, shaping their perceptions, behaviors, and decision-making processes.^12^ At the interface between information, marketing and entertainment, content on these platforms can amplify both positive health messages and harmful misinformation, creating a dynamic environment where health-related information is influenced by trending narratives.^12^ During the COVID-19 pandemic, social media played an important role in sharing key data rapidly across geographical boundaries to health workers and the public. However, social media also contributed to the rapid spread of misinformation, causing public skepticism and the anti-vaccine movement.^13^ Similarly, emerging evidence suggest that social media platforms significantly influence the uptake of sun safety information among younger populations, potentially influencing their adherence to sunscreen recommendations.^14-17^

While prior studies have explored general sun-exposure and sun-protection behaviors on social media, the impact of anti-sunscreen sentiments, specifically on youth-dominated platforms like TikTok, remains underexplored. The aims of this study are 1) to analyze the content and format of anti-sunscreen sentiments, reflected by the #nosunscreen hashtag, on TikTok, 2) to inquire into common reasons for being anti-sunscreen, and 3) to provide insights regarding the impact of these narratives on sun safety behaviours among younger individuals. These findings could promote evidence-based sun safety practices, ultimately contributing to the creation of tailored skin cancer prevention strategies by leveraging digital platforms that specifically target these high-risk populations.

## Methods

### Data Collection

A collection of TikTok videos tagged with #nosunscreen was conducted in July 2024. The search was performed in an incognito browser and without any linked personal account. A sample size calculation was not performed, all videos relevant to the analysis were included. All TikTok videos were included until consensus among the researchers was reached that the videos no longer demonstrated relevance to the hashtag employed. Videos were excluded if they were duplicates, if the video or audio was unavailable, if they were in a language other than English, or if they were not related to sunscreen.

Prior to data collection, reviewers were trained using a sample set of videos. Data was then systematically collected by two independent reviewers using a standardized data collection tool. Any disagreements in evaluations were resolved through group discussion, ensuring consensus among the reviewers. The following information was collected for each video:

1) Video characteristics: username, TikTok handle, user profile (individual or organisation), caption, hashtags, date of upload, video length, language of the video, and whether videos were part of a playlist.
2) Engagement levels: number of followers, views, likes, comments, saves, and shares.
3) Consumer details: presence of sponsorships, product recommendations and account endorsements.
4) Audio-visual production features: insertion of pictures or videos, presence of written text on screen, incorporation of a duet (defined as a response video where two videos play side by side) or stitch (defined as a response video where one video follows another), filming location, and use of music and/or audio memes.
5) Demographic information on the creator (when available): age, gender, and ethnicity, including visible minorities, defined by Statistics Canada as “persons, other than Aboriginal peoples, who are non-Caucasian in race or non-white in colour.”^18^
6) Content:
  a-temporal dynamic (whether the video was filmed in real time or was referring to the past).
  b-display of explicit emotions, self-derision, sarcasm and/or irony.
  c-users’ display of sunburn.
  d-opinions on wearing sunscreen, reasons for not wanting to wear sunscreen and self-reflection on their behaviours.
  e-intended audience and public service announcement (when available).

### Thematic Analysis and Sunscreen Sentiment

Video themes were extracted both deductively and inductively, based on notes taken by the reviewers during the viewing process. Themes collected are described in Table 1.

**Table 1.** Definition of Anti-Sunscreen Themes

Theme	Definition
Alternative Sun Protection	Refers to alternative sun protection methods used by individuals such as natural home remedies that people may believe are equivalent to sunscreen or even the use of tanning oils or diets.
Benefits of Natural Sun Exposure	Refers to the health advantages derived from sun exposure. They include physiological effects such as cutaneous vitamin D synthesis which plays an important role in musculoskeletal health, cardiovascular health, and immune function. Furthermore, psychological benefits such as mood improvement through increased serotonin levels and the regulation of circadian rhythms.^19^
Cultural or Skin-Type Beliefs	Refers to individuals with darker skin complexion not applying sunscreen because of the assumption that they will not burn.
Desire to Tan	Refers to individuals purposely not applying sunscreen and exposing their skin to the sun with the goal of achieving a darker complexion.
Anti-Regulation Sentiments	Refers to a lifestyle in which individuals prioritize personal freedom and lived with fewer rules and constraints.
NPOS	Stands for Never Putting On Sunscreen
Personal Preferences/Trendy Effect	Refers to the influence of current social trends or personal preferences, especially the impact of tanning on one’s appearance.
Sun coating	Refers to the practice of building a base tan to create a protective layer against further sun exposure.
Sunpoison	Refers to the state of having a severe sunburn that resembles an allergic reaction.
Sunscreen Associated with Skin Cancer	Refers to the belief of sunscreen being associated with any type of skin malignancy such as basal cell carcinoma, squamous cell carcinoma or melanoma.
Sunscreen Blocks Vitamin D	Refers to the belief of sunscreen being associated with the prevention of ultraviolet (UV) radiation from reaching the skin, which helps the body to produce vitamin D.
Sunscreen as Harmful	Refers to concerns raised about ingredients in sunscreens that may negatively impact health such as benzene.
Sunscreen as Unnecessary	Refers to the viewpoint that sunscreen does not protect against the sun.

### Statistical Analysis

Descriptive statistics were used to report the collected data. Spearman’s correlation coefficient, and Phi coefficient were computed to measure the strength of the relationship between variables. As established by Akoglu,^18^ the Phi coefficient can be interpreted as follows: <0.1 weak, >0.1 moderate, >0.15 strong and >0.25 very strong; and the Spearman coefficient as follows: >0.5 moderate/strong, >0.7 strong/very strong. The Pearson chi-squared test was performed to test statistical significance between binary variables (at alpha 0.05). All the analyses were performed using STATA version 18.0.

### Ethical Considerations

Ethics review was not required, as all the material used was in the public domain and volunteered to TikTok by the creators of the videos. No personal identifying information was collected for this study. All personally identifiable information (such as creator usernames) has been omitted from the manuscript and is not considered relevant to its analysis. The reporting of this study conforms to STROBE guidelines.^20^

## Results

### Data Collection

A total of 401 videos were identified. Of these, 53 were excluded as the video or audio were unavailable, 23 because they were not related to sunscreen, and 4 because they were in another language than English. As a result, 321 videos were analysed.

### Video & Creator Characteristics

Of the videos analysed, 311 (97%) were posed by an individual handle and 291 (91%) were posed by unique TikTok users. All videos had an associated caption and 9% included hashtags (which were captured to assess whether creators were making active efforts to align their content with that of others). The videos were uploaded onto the platform between January 2020 to July 2024, with the majority (38%) posted in 2023 (Table 2). The average video length was 25 seconds (SD 3 seconds), the shortest video being only 4 seconds and the longest one being almost 10 minutes.

**Table 2.** Year Posted

Year posted	n (%)
2020	19 (5.9)
2021	33 (10.3)
2022	66 (20.6)
2023	123 (38.3)
2024	80 (24.9)

A person belonging to a visible ethnic minority (defined by Statistics Canada as “persons, other than Aboriginal peoples, who are non-Caucasian in race or non-white in colour”)^21^ was present in 61 videos (19%). The majority of video creators were female (78%), and almost half (48%) were believed to be younger than 25. Creator characteristics are listed in Table 3.

**Table 3.** Creator Characteristics

Characteristic	n (%)
Female	250 (78%)
Male	71 (22%)
Younger than 25 years old	155 (48%)
Visible ethnic minority	61 (19%)

### Engagement

The mean number of followers per creator was 90,054 (SD 324,640; range 31 - 2,700,000). The mean number of viewers per video was 73,121 (SD 408, 246), whereas the mean number of likes was 5,250 (SD 31,772). Commenting was enabled in 312 (97%) of videos with a mean of 51 comments per video (SD 203; range 0 to 2448). The mean number of saves and shares were 490 (SD 4,289) and 222 (SD 1,569), respectively. Engagement metrics are listed in Table 4.

**Table 4.** Engagement

Engagement	Mean (SD)	Min-max
Followers	90.054 (324.640)	31-2.700.000
Views	73.121 (408.246)	220-5.600.000
Likes	5250 (31.772)	15-380.800
Comments	51 (203)	0-2448
Saves	490 (4289)	0-71.400
Shares	222 (1569)	0-19.000

### Video Production Features

Overall, 145 videos (45%) incorporated music and 95 (30%) incorporated an audio meme. No video was part of a playlist. A visual aid was incorporated in 64 cases (20%). Half of these were presented in Graphics Interchange Format, commonly referred to as GIFs, which are short, animated clips often used to convey emotions or humour in digital conversations. Additionally, 8 videos explicitly displayed the recorded UV index. The majority of videos (240, 75%) included written text on screen. Only 9 videos (3%) were a duet (defined as a dual-screen response where the creator responds to another video while both videos appear side by side), and 6 videos (2%) were a stitch (defined as a video response where the video being responded to plays first, with the response following). Videos were mainly filmed at home (57%), with the creator’s bedroom being the most frequent location. Additionally, 101 videos (31%) were recorded in an outdoor setting, with the most common identifiable locations being the creator’s backyard (14%) or the beach (7%). 147 videos (46%) were filmed in real time, and 62 videos (19%) were explicitly referring to past events. Finally, overt corporate sponsorships were only present in a minority (4%) of TikToks, present in either the caption and/or video itself. Of these, 11 recommended products, while 1 endorsed an individual. Only 3 videos promoted the creator’s own account (1%).

Visibly burnt skin, defined as erythematous dry peeling and/or blistering of the cutaneous integument, was displayed in 142 videos (44%). Finally, 61 videos (19%) displayed a “desirable tanned” skin, defined as a “glowing healthy golden/bronzed tone”. Very few videos (3%) incorporated a photoaging filter, identified by the display of hyperpigmentation, drying, thinning, and loss of firmness of the skin. Production features and visuals are summarized in Table 5.

**Table 5.** Production Features and Visuals

Production features	n (%)
Music present	145 (45.2)
Audio meme present	95 (29.6)
Visuals (inserted pictures/videos)	64 (19.9)
UV	8 (2.5)
GIF	32 (8.9)
Ageing/skin filter use	11 (10.0)
Sunburn displayed	142 (44.2)
Tan displayed	61 (19.0)
Display of emotion	30 (9.4)

### Content: Sunscreen Sentiment

A personal opinion on wearing sunscreen was explicitly stated in in 136 videos (42%), whereas an additional 78 videos (24%) implicitly suggested a viewpoint. Creators provided justification for their viewpoint for not wearing sunscreen in 157 videos (49%). Of those, only 18% engaged in self-reflection on their behaviours, admitting that they should have worn sunscreen. Only a minority 32 videos (9%), included a public service announcement.

Among the creators that justified their anti-sunscreen stance, several reasons were provided, which were categorized into distinct themes and summarized as proposed anti-sunscreen sentiments (Table 6; refer to Table 1 for stance definitions). The most commonly referred to reasons were: personal preferences related to trends, belief in the benefits of natual sun exposure, and the belief that sunscreen is unnecessary. It was common to have more than one reason used as a justification. Specific terminology such as NPOS (“never putting on sunscreen”) was mentioned in 2 videos, “sun poison” was referred to in 6 videos, and the “anti-regulation sentiments” theme was used in 6 videos. “Never putting on sunscreen” is a phrase used to signal anti-sunscreen sentiments on social media. “Sun poison” terminology appeared in videos where creators admitted to enduring sun damage but did not admit that sunscreen could have prevented it.

**Table 6.** Sunscreen Sentiments

Sunscreen sentiments	Overall (n=321)		Can you identify if this video is for or against using sunscreen?			
			Yes (n=156)		No (n=165)	
	n	%	n	%	n	%
Proposed anti-sunscreen stances						
Personal Preferences/Trendy Effect	26	8.1	14	9.0	12	7.3
Natural Sun Exposure Benefits	22	6.9	15	9.6	7	4.2
Sunscreen as Unnecessary	18	5.6	5	3.2	13	7.9
Alternative Sun Protection	17	5.3	9	5.8	8	4.8
Desire to tan	17	5.3	9	5.8	8	4.8
Sunscreen as Harmful	16	5.0	7	4.5	9	5.5
Sunscreen Blocks Vitamin D	8	2.5	2	1.3	6	3.6
Sunscreen Associated with Skin Cancer	7	2.2	3	1.9	4	2.4
Sunpoison Associated with Sunscreen	6	1.9	1	0.6	5	3.0
“Live free or die”	6	1.9	1	0.6	5	3.0
Cultural or Skin-Type Beliefs	5	1.6	3	1.9	2	1.2
NPOS	2	0.6	1	0.6	1	0.6
Any stances	124	38.6	55	35.3	69	41.8
It is cool/funny to be burnt	69	21.4	26	16.7	43	26.1
Explicit Regret	24	7.5	13	8.3	11	6.7
Creator admits they should have worn sunscreen	57	17.8	48	30.8	9	5.5
Position stated explicitly	136	43.3	96	61.5	40	24.2
Position stated implicitly	78	24.2	59	37.8	19	11.5

Interestingly, 136 videos (42%) conveyed sarcasm and/or irony in their tone. This was explicitly expressed by the creator in 51 videos (16%). The presence of self-derision, defined as the creator’s ability to “make fun of themselves” for not having worn sunscreen, was noted in 78 videos (24%). The sentiment of “being cool” for not having worn sunscreen was present in 69 videos (22%). A positive and/or negative display of emotion was only judged present in 30 videos (9%), with regret identified in 24 videos (7%).

### Statistical Correlations

A strong to very strong positive monotonic association was noted between views, likes, comments, and shares, with each pair of variables increasing together (spearman correlation coefficient between 0.63 and 0.84). The monotonic association between comments and the other engagement variables was only moderately positive (spearman correlation coefficient is between 0.53 and 0.59).

Among videos where an anti-sunscreen sentiment was clearly identified (n = 156), a strong statistically significant association was observed between the various anti-sunscreen justifications and videos that were not intended as public service announcements (Chi-square test, p<0.001). There was no statistically significant association between the various anti-sunscreen justifications and any targeted demographic population, although the results approached significance (p=0.055).

Additionally, there is no to very weak positive monotonic association between these various anti-sunscreen stances and the different variables of engagement, namely views, likes, comments, saves and shares, with each pair of variables increasing together (spearman correlation coefficient is between 0.00 and 0.10).

Moreover, a strong statistically significant association was observed between some of the anti-sunscreen stances and the videos where creators did not utilize self-derision (Chi-square test, p<0.001). There was also a strong statistically significant association observed between some of the anti-sunscreen stances and the videos where creators failed to express regret for not wearing sunscreen (Chi-square test, p<0.001). A strong statistically significant association was observed between the previously described anti-sunscreen stances and the videos where the sentiment of “being cool” was vehiculated (Chi-square test, p=0.024). Videos expressing sentiment of “being cool” had a very strong relationship with the desire to tan (ϕ=0.26), as well as a had a strong relationship with personal preferences (ϕ=0.16) (Table 7). Videos conveying sarcasm and/or irony had a strong relationship with the beliefs that natural sun exposure had more benefits than sunscreen (ϕ=0.25), that sunscreen is harmful (ϕ=0.19), and that alternative form of protection are deemed acceptable (ϕ=0.22). Videos utilizing self-derision had a strong relationship with the beliefs that natural sun exposure had more benefits than sunscreen (ϕ=0.21), and that alternative forms of protection are deemed acceptable (ϕ=0.16). There was no statistically significant association between the creator’s age and gender with anti-sunscreen beliefs (Chi-square test, p=0.441 and p=0.187, respectively).

**Table 7.** Correlation Analyses

Correlation between column and row variables/PHI (ϕ) coefficient	Public service announcement to wear sunscreen	Sarcasm and/or irony conveyed	Self-derision for not wearing sunscreen	“Being cool/funny” for not wearing sunscreen	Creator admits they should have worn sunscreen	Age demographic targeted
Alternative Sun Protection	0.12	**0.22**	0.16	0.04	0.17	0.01
Benefits of Natural Sun Exposure	0.16	**0.25**	**0.21**	0.09	**0.22**	0.10
Desire to tan	0.05	0.05	0.04	**0.26**	0.11	0.08
Personal Preferences/Trendy Effect	0.15	0.05	0.15	0.16	**0.21**	0.12
Sunscreen as Harmful	0.10	0.19	0.14	0.10	0.08	0.13
Sunscreen as Unnecessary	0.09	0.06	0.04	0.02	0.12	0.06
Sunscreen Blocks Vitamin D/Cultural Reasons/Skin Cancer/NPOS/	0.07	0.09	0.12	0.15	0.12	**0.24**

Very strong relationships are in bold font.

## Discussion

This study provides a content analysis of TikTok videos tagged #nosunscreen. Using descriptive, thematic and statistical analysis, it demonstrates that 1) TikTok is a digital setting in which anti-sunscreen sentiments are openly discussed, 2) that the use of humour is a key mechanism through which such sentiments are communicated, and 3) that common reasons for not wanting to wear sunscreen range from personal preferences, to perceptions of sunscreen as unnecessary or harmful, to the perceived benefits of natural sun exposure. This study also highlights the relevance and potential impact of perceived social media trends. This study demonstrated that there is a rising trend in anti-sunscreen sentiment on TikTok, with a growing number of videos published under #nosunscreen from 2020 to 2024. The creators of these videos were primarily individuals, many of whom use different techniques to support their anti-sunscreen stance. Over half the videos featured a form of humorous expression, with some who presented being sunburnt as “being cool” to normalize their behavior. In contrast, some avoided using irony and sarcasm, using structured, well-developed arguments or personal beliefs to explicitly justify their anti-sunscreen behavior and persuade viewers. Many creators highlighted the perceived benefits of sun exposure as a rationale for not wearing sunscreen with some claiming that sunscreen blocks vitamin D and others that sunscreen contains harmful ingredients. The desire to achieve a tan or the use of a substitute sun protection method were also other common reasons cited by TikTok creators for avoiding sunscreen.

Melanoma rates have increased among youth^1-4^ and sun protection behaviours among this population have regressed.^10,11^ Social media content poses the risk of influencing user opinions, a phenomenon made evident by the rise of the anti-vaccine movement during the COVID-19 pandemic. As such, it is imperative to attend to the ways that social media might influence other crucial health behaviours such as melanoma prevention. The TikTok platform was the ideal setting for this study due to its young audience and user base, its engaging and interactive user experience, and the highly consumable and spreadable nature of its content. TikTok, a popular and rapidly growing social media platform, stands out due to its focus on short, engaging video content that fosters a highly interactive environment. TikTok videos tend to be shorter, with our study demonstrating an average length of 25 seconds. This is significantly shorter than YouTube videos, which tend to be 15-to-20-minutes in length.^22,23^ This platform also became a hub for viral trends and user-generated material, setting it apart from other social media platforms by emphasizing spontaneity, creativity, and community engagement. These characteristics played a pivotal role in shaping the dynamics observed in this study, making TikTok the ideally suited social media platform to assess the #nosunscreen trend. The platform’s high engagement around specific hashtags is further demonstrated in a recent study that assessed the #sunburnchallenge and #tanningbedchallenge,^24^ showing similar results.

The forms of engagement received by the TikToks analyzed in this study are key to understanding their audience. Where follower count, likes, shares, and saves were relatively high in number, we observed a low number of average comments per video, suggesting low levels of engagement effort. Conversely, social media research in the context of metastatic breast cancer^25^ found that higher numbers of comments on social media posts are associated with materials which are highly emotional and sentimental in nature, such as posts about recurrence and end of life care, and that these materials garner higher engagement efforts by followers. As such, the lack of comments found in this study is a potential signal that anti-sunscreen TikToks may not be considered as highly sentimental or sensitive videos by their audiences, and that comment sections may not be the source to draw on in efforts to understand these videos’ reception and perceieved meaning.

The lack of sensitivity around melanoma observed in these videos suggests that, unlike in highly personal videos about breast cancer, the harsh realities and severity of melanoma are made abstract and trivialized. These are highly shareable videos that seem to circulate with ease, and these qualities, paired with the light and at times flippant approach to the threat of melanoma, necessitate the development of novel approaches to understanding the ways that youth are exposed to and influenced by anti-sunscreen messaging online. It is also important to consider the possibility that, in the context of these videos, there could be no perceieved link between sustaining a sunburn and the future possibility of developing melanoma. There is a possibility that many these videos are rooted in concerns over appearance rather than matters of health. Indeed, research suggests that appearance is a crucial element in mitigating sunscreen use: where the desire for a tanned, “glowy” appearance is used to justify not wearing sunscreen, as is the residue that sunscreen leaves behind on skin, it was also found that appearance-based approaches to sunscreen use were more successful than health-based ones.^16^

The minimal statistical association between anti-sunscreen stances and engagement variables suggests that there are many unknowns as to what is precisely responsible for the spread of misleading information about sunscreen use as melanoma prevention. The lack of statistical association between anti-sunscreen sentiments and engagement variables could be a result of TikTok’s algorithm prioritizing certain types of content; it could also due to the possibility that it is other video qualities, such as audio trends or length, which garner higher view counts and not necessarily their message concerning sunscreen. However, the strong association of anti-sunscreen stances with videos that lacked any self-derision on the part of the creator suggests that these individuals are firm in their beliefs, and whether or not it is their intent to influence their audience’s opinions, there is a clear unmitigated risk of spreading dangerous misinformation.

Tone (including elements of irony, sarcasm, and self-derision) was a significant element of anti-sunscreen messaging on TikTok. In our study, more than half of the videos featured a form of humorous expression, a tendency consistent with what has been observed in other content domains on TikTok, such as vaccine skepticism^26^ and mental health following traumatic life events.^27^ We found that 42% of videos conveyed some level of sarcasm or irony, and that these videos held a strong correlation with the beliefs of the perceived benefits of natural sun exposure, that sunscreen is harmful, and the consideration of alternative forms of sun protection as acceptable. Similarly, videos which included self-derision held a strong correlation with belief in the perceived benefits of natural sun exposure and the consideration of alternative forms of sun protection as acceptable. TikTok’s user interface encourages viewers to imitate or replicate content; if something is humorous or relatable, it is more likely to be replicated and rapidly spread.^28^ Additionally, humour is found to provide a sense of reassurance, when something is funny or ridiculous it is perceived as less threatening, thereby becoming a tactic for the spread of misinformation.^29-31^ This was confirmed in our study, where a creator laughs throughout a conversation with his mother who tells him he looks like a cooked lobster to which he responds with #skincancer before he bursts out laughing. By joking about the threat of skin cancer, this creator downplays the seriousness of this disease. In another video, a creator writes, “Hey mom look I am tanned” while showing us her clearly burnt back. The comment section is filled with laughing emojis supporting this idea that a sunburn is funny rather than a sign of potential skin damage, reflecting the potential ways that engagement intensity may support the formation of echo chambers online. As such, that many TikToks might be considered humorous is not coincidental; rather, it is an integral part of how and why these videos spread with such ease.^28^ Indeed, the use of humour to “tone down” serious subject matter on TikTok is not limited to the examples of skin cancer that this study has analyzed; other studies have identified instances of serious matters being turned humorous, which demonstrate humour as a foundational element of how people communicate on TikTok.^32^ Popular sounds and meme creation are also elements that aid in the spread and circulation of TikTok videos.^28^

Misinformation and/or misleading information is not always obvious: its recognition requires an understanding of how false ideas circulate. Research has demonstrated how misinformation creators plant small “seeds of doubt” or target emotions to build trust and convince their viewers.^33^ For example, during the COVID-19 pandemic some creators would plant seeds about forms of corruption to create doubt in their viewers about COVID and the vaccine.^34,35^ In the case of benzene, creators are not only planting seeds of doubt, but they are also targeting people’s emotions by using words like “cancer.” A commonly cited reason by TikTok creators for avoiding sunscreen is the belief that it is harmful. Over 11% of the anti-sunscreen videos in this study demonstrate the beliefs that sunscreen is either harmful or unnecessary. Beyond concerns of vitamin D production, videos in our study highlighted the potential health risks of chemicals found in sunscreen. Indeed, the discovery of benzene, a known human carcinogen, in certain sunscreen products created significant turmoil, sparking widespread concern about the safety of sunscreen.^36,37^ Despite these products being removed from the market, the discovery sparked a discussion around the balance between protecting oneself from harmful ultraviolet radiation while avoiding carcinogens, both of which are crucial public health concerns.^36,37^ While concerns about the safety of sunscreen ingredients like benzene have fueled skepticism, it is essential to remember the benefits of sun protection against the risks of ultraviolet radiation. As such, this study’s findings align with previously recognized forms of misinformation circulation. Combined with their often humorous or sarcastic nature, the “seed of doubt” quality of many of these TikToks may serve to disguise them as lighthearted and interesting, rather than harmful and misleading.

TikTok drives a younger user population, with studies suggesting that 41% of TikTok users are between the ages of 16 and 24.^38^ Given the rising incidence of melanoma among adolescents,^1-4^ these demographic patterns have important implications. A study showed that the most common threat, encountered daily and weekly on social media, was misinformation.^39^ Adolescents are vulnerable to this threat because they are crossing a period of developmental sensitivity due to social, biological, and psychological development. One of the most significant areas of sensitivity is related to social interaction because of peer influence and self-perception.^40^ Chen and colleagues found that some college students were motivated to share misinformation for reasons related to self-expression and social interaction, because the information is a good topic of conversation or is interesting.^41^ In addition, young people’s ability or even will to detect misinformation is inadequate. A study showed that more than half of the young people consulted never or rarely try to find if the information they encounter online is true or not, another showed that 31% of kids aged 10 to 18 have shared online at least one news story that they later found out was inaccurate or fake.^42,43^ The vulnerability of this population, their tendency to share engaging content as well as their inability or lack of effort to detect misinformation creates a dangerous environment where misinformation related to sun protection disguised as humorous or factual propagates quickly. As such, the informational vulnerabilities at play in this online environment create the conditions in which young people are particularly susceptible to engaging with misleading and harmful portrayals of sunscreen.

Several limitations should be noted for this study. First, there is potential selection bias, as only a limited subset of TikTok videos under the #nosunscreen hashtag were included. The videos were selected until they were deemed to be no longer relevant to the topic of sunscreen. The analysis was limited to English-language videos, potentially excluding relevant content from other languages and cultural perspectives. Second, an interpretation bias should be considered given the very subjective nature of the data analyzed. Indeed, personal perspectives influence the interpretation of humorous content, particularly when it includes irony, sarcasm, self-derision, or other subtle linguistic nuances. Throughout the study, we observed that some videos were becoming unavailable, likely as a result of content moderation policies enforced by the social media platforms. This highlights the study’s cross-sectional design, where only a snapshot of the content available is being captured. Given the rapidly evolving nature of social media, the videos analyzed may not accurately represent all anti-sunscreen content on TikTok over time. The descriptive nature of the study, which was conducted at a single point in time, also limits our ability to infer causality. Future research should address these gaps by conducting longitudinal studies to observe changes over time. It should also aim to develop and validate research methodology specific to the dynamic, ever-changing reality of social media materials. Another limitation of our study is the inability to measure the direct impact of viewed content on individual behavior. Although engagement metrics were recorded, they do not offer clear insights into the impact on viewer’s health-related decisions. Future research should explore the effects of viewing and engaging with anti-sunscreen videos on TikTok on user behavior. Furthermore, it was beyond the scope of our research to evaluate the validity and accuracy of the information shared in those videos.

## Conclusion

This study enhances our understanding into the role TikTok plays as a source of health information, particularly in promoting anti-sunscreen sentiments. Despite the growing body of evidence supporting the use of sunscreen as a critical preventative measure, the prevalence of misinformation surrounding sunscreen use on social media is concerning. TikTok videos under the #nosunscreen hashtag often promote misleading narratives on sun safety. These messages, frequently delivered through humour, also diminish the perceived importance of sunscreen use. As social media continues to shape public health perceptions, it is essential for public health professionals to actively engage with platforms like TikTok to provide evidence-based information in formats that resonate with younger audiences. Ultimately, the strategic dissemination of credible health information through digital platforms like TikTok is crucial for developing effective public health strategies. It is essential to gain a better understanding of how anti-sunscreen sentiments are disseminated through social media and the ways in which such messaging targets those who may already be doubtful or vulnerable to influential content. Specific next steps toward leveraging digital platforms in this context should include a large scale study that investigates how young people assess the health information they engage with on social media, and how they perceive information to be credible or not. Such findings could directly inform and shape public health messaging strategies around sun safety among youth.

## Data Availability

All data generated or analyzed during this study are included in this published article.*

## Appendix

### Abbreviations

NPOS: Never putting on sunscreen
GIF: Graphics interchange format
UV: Ultraviolet.
